# Identification of biomarkers and construction of a microRNA‑mRNA regulatory network for clear cell renal cell carcinoma using integrated bioinformatics analysis

**DOI:** 10.1371/journal.pone.0244394

**Published:** 2021-01-12

**Authors:** Miaoru Han, Haifeng Yan, Kang Yang, Boya Fan, Panying Liu, Hongtao Yang

**Affiliations:** Department of Nephrology, First Teaching Hospital of Tianjin University of Traditional Chinese Medicine, Tianjin, P. R. China; University of Calgary, CANADA

## Abstract

With the recent research development, the importance of microRNAs (miRNAs) in renal clear cell carcinoma (CCRCC) has become widely known. The purpose of this study is to screen out the potential biomarkers of renal clear cell carcinoma (CCRCC) by microarray analysis. The miRNA chip (GSE16441) and mRNA chip (GSE66270) related to CCRCC were downloaded from the Gene Expression Omnibus (GEO) database. After data filtering and pretreating, R platform and a series of analysis tools (funrich3.1.3, string, Cytoscape_ 3.2.1, David, etc.) were used to analyze chip data and identify the specific and highly sensitive biomarkers. Finally, by constructing the miRNA -mRNA interaction network, it was determined that five miRNAs (*hsa-mir-199a-5p*, *hsa-mir-199b-5p*, *hsa-mir-532-3p* and *hsa-mir-429*) and two key genes (*ETS1* and *hapln1*) are significantly related to the overall survival rate of patients.

## 1 Introduction

Renal cell carcinoma is a common malignant tumor, which accounts for 90% of all renal tumors [1]. According to the characteristics of histopathology and molecular biology, renal cell carcinoma can be divided into four parts, including clear renal cell carcinoma, papillary renal cell carcinoma, chromophobe renal cell carcinoma and Bellini collecting duct carcinoma. Among them, 85% are clear renal cell carcinoma (CCRCC) [2]. At present, it is often diagnosed according to the clinical symptoms, imaging, renal biopsy, etc. However, many small renal mass (RMs) patients have no symptoms until the late stage of the disease, 30% of them have had distant metastasis when they are diagnosed as CCRCC [3]. Therefore, a sensitive and accurate diagnosis method of CCRCC is urgently needed. Recent studies have found that microRNAs (miRNAs) plays an important role in the pathogenesis of renal cancer [4,5]. Phosphatase and tensin homolog (*PTEN*) is one of the most extensively studied tumor suppressor genes related to tumor metastasis. Studies have shown that the expression of *miR-22* is down-regulated in the tumor tissue of CCRCC patients. In addition, in order to inhibit the growth, migration and invasion of tumor cells [6]. *miR-22* may directly target *PTEN* in renal cell carcinoma. *MiR-21* is involved in the fine-tuning of von Hippel Lindau tumor suppressor (VHL) expression, and VHL tumor suppressor gene is involved in the regulation of hypoxia response through the regulation of hyperxia inducible factors (HIFs) [7] VHL-HIF signaling pathways play an important role in the pathogenesis of CCRCC. In addition, *miR-155* [8], *miR-185* [9] play a key role in the prognosis of renal cell carcinoma. In this study, the common data sets of GSE16441 and GSE66270, the differential expression of miRNA chip and mRNA chip, and the functional enrichment and pathway enrichment of different genes were analyzed. Finally, the miRNA- mRNA regulatory network of CCRCC was constructed to identify and screen out the specific and highly sensitive prognosis biomarkers related to CCRCC. It include five miRNAs (*hsa-mir-199a-5p*, *hsa-mir-199b-5p*, *hsa-mir-532-3p and hsa-mir-429*) and two key genes (*ETS1* and *HAPLN1*). However, the regulatory network between miRNA and mRNA is very complex and needs to be studied further.

## 2 Materials and methods

### 2.1 MiRNA and mRNA expression profiles of CCRCC

The GeneExpression Omnibus (GEO) database http://www.ncbi.nlm.nih.gov/geo/ served as an international public data archive, distributes high-throughput gene expression and other functional genomic datasets free of charge [10]. miRNA and mRNA micro-array datasets were downloaded from the GEO database under the accession number GSE16441 and GSE66270. The miRNA data set GSE16441 includes 18 normal control samples and 18 clear renal cell carcinoma samples (S1 Table). All samples were detected by the platform GPL8659 Agilent human miRNA microarray rel12.0. The gene expression profile GSE66270 includes 17 normal control samples and 17 clear renal cell carcinoma samples (S2 Table). The chip platform is GPL570 [HG-U133_ Plus_ 2] Affymetrix Human Genome U133 Plus 2.0 Array.

### 2.2 Data processing and differentially expressed RNAs

In https://www.ncbi.nlm.nih.gov/geo/query/acc.cgi?acc=GSE16441 download the series matrix file (s) and GPL8659 Agilent human miRNA microarray rel12.0 platform files. According to the corresponding platform annotation information, the samples were grouped by Perl software, and the probe ID was converted into gene symbols. Also in https://www.ncbi.nlm.nih.gov/geo/query/acc.cgi?acc=GSE66270 Download series matrix file (s) and GPL570 [HG-U133]_ Plus_ 2] Affymetrix human genome U133 Plus 2.0 array platform file, the samples were grouped in the same way, and the probe ID was converted into gene symbol. R / Bioconductor's "limma" package was used to identify the differentially expressed miRNAs (DEmiRs) in individual studies. Initially Benjamin Hochberg (BH) corrected p value was to be < 0.05 and |log2 fold change (FC) | was set to be > 1 (S3 Table). Using the same method, set the adjusted value of Benjamin Hochberg (BH) was adjusted to be < 0.05 and | log2 fold change (FC) | was adjusted to be > 2 to identify the differentially expressed mRNAs (DEGs) (S4 Table). Finally, the volcano map was drawn using R software.

### 2.3 Functional and pathway enrichment analysis of miRNA with differential expression

In order to further understand the biological function of DEmiRs, the gene ontology (GO) and the Kyoto Encyclopedia of genes and genomes (KEGG) pathway enrichment of DEmiRs were studied through funrich3.1.3 [11]. GO enrichment analysis included "biological process" (BP), "cellular component" (CC) and "molecular function" (MF). There was statistical significance in setting P < 0.05.

### 2.4 PPI network construction and module selection

Upload DEGs to search tool for retrieval of interacting genes (string) database [11] (https://string-db.org/), build PPI network, and set the confidence score ≥ 0.95 (S5 Table). Then import the network into Cytoscape_3.2.1 [12] software for visualization, using the Molecular Complex Detection (MCODE) plug-in to filter the DEGs module (degree cutoff = 2, k-core = 2, max. depth = 100 and node score cutoff = 0.2). Then according to the score value, the first four modules are selected for subsequent analysis. In order to further understand the biological function of DEGS in the module, the R / cluster Profiler [13] software package was used to perform Gene Ontology (GO) enrichment analysis and Kyoto Encyclopedia of Genes and Genomes (KEGG) pathway enrichment analysis on DEmiRs. P < 0.05 was the threshold. R / ggplot2 [14] software package was used for visualization of GO enrichment analysis, and the first ten item parameters are limited for display. The R / goplot [15] software package was also used to visualize the enrichment analysis of KEGG pathway. The first six pathway entries were defined for display.

### 2.5 miRNA-mRNA Interactions analysis and visualization

Similarly,funRich3.1.3 was used to predict the target genes of DEmiRs, and we obtained a total of 2070 target genes (S6 Table), then we used Practical Extraction and Report Language (Perl v5.30.0) [16] to integrate the target genes, DEmiRs and DEGs data to identify the commonly Genes in the predicted target genes and DEGS. Finally, we obtained the regulatory network of miRNA target genes and DEGS (S7 Table). Using Cytoscape_3.2.1 software to visualize the regulatory network. This regulatory network includes a total of 69 nodes and 66 edges (Fig 4).

### 2.6 Survival analyses

Gene Expression Profiling Interactive Analysis (GEPIA, http://gepia.cancer-pku.cn/index.html) based on TCGA database [17] was used to conduct online analysis of DEGS in the regulatory network. Kaplan Meier survival chart was used to compare the overall survival of the two groups of patients. Group cutoff was set as median, consistency interval was 95%, and log rank p value < 0.05 was statistically significant. The central genes were identified from the predicted target genes for subsequent topological analysis, and degree was used as the screening condition for nodes with high connectivity.

## 3 Results

### 3.1 Identification of differentially expressed miRNAs and mRNAs in CCRCC

**Fig 1:**
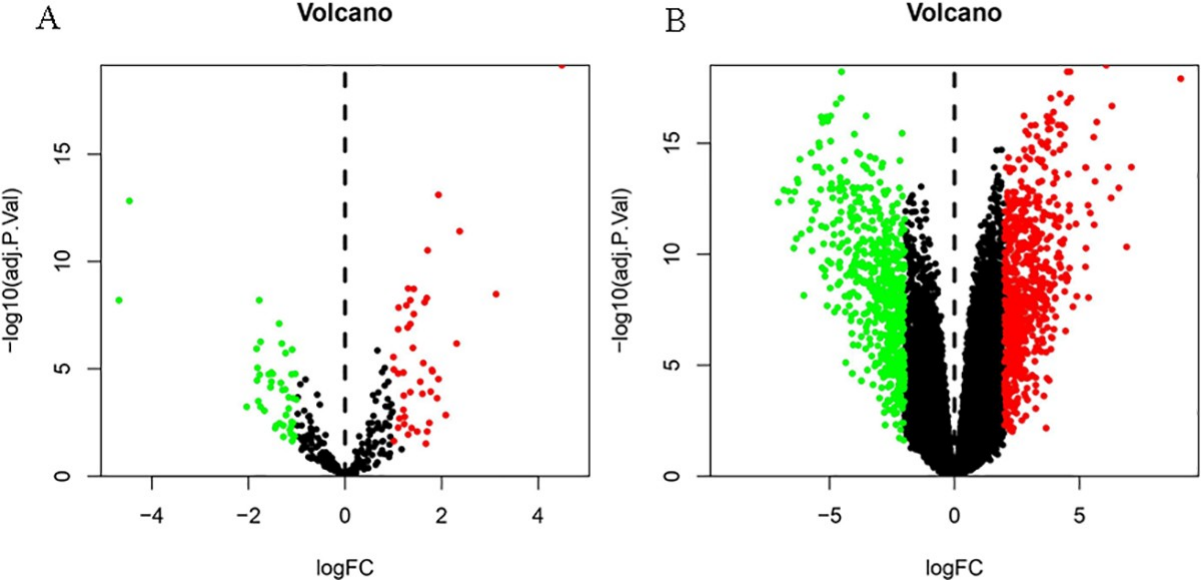
Volcano plot of DEmiRs and DEGs. A, Volcano plot of differentially expressed miRNAs. The red dot represents upregulated miRNAs and the green dot represents downregulated miRNAs. B, Volcano plot of differentially expressed mRNAs. The red dot represents upregulated mRNAs and green dot represents downregulated mRNAs.

After searching the appropriate datasets in GEO database according to the eligibility criteria, two datasets (GSE16441 and GSE66270) regarding CCRCC were chosen as discovery sets. By setting the threshold of adjust P < 0.05 and | logfc | > 1.0.A result of 90 DEmiRs was obtained from GSE16441 data set, including 45 were up-regulated and 45 were down-regulated. (Fig 1A). By adopting the same method and setting threshold of adjust P < 0.05 and | logfc | > 2.0, and a total of 1309 DEGs were obtained from GSE66270 data set, of which 718 were up-regulated and 591 were down-regulated.(Fig 1B).

### 3.2 Enrichment analysis of differentially expressed miRNAs

**Fig 2:**
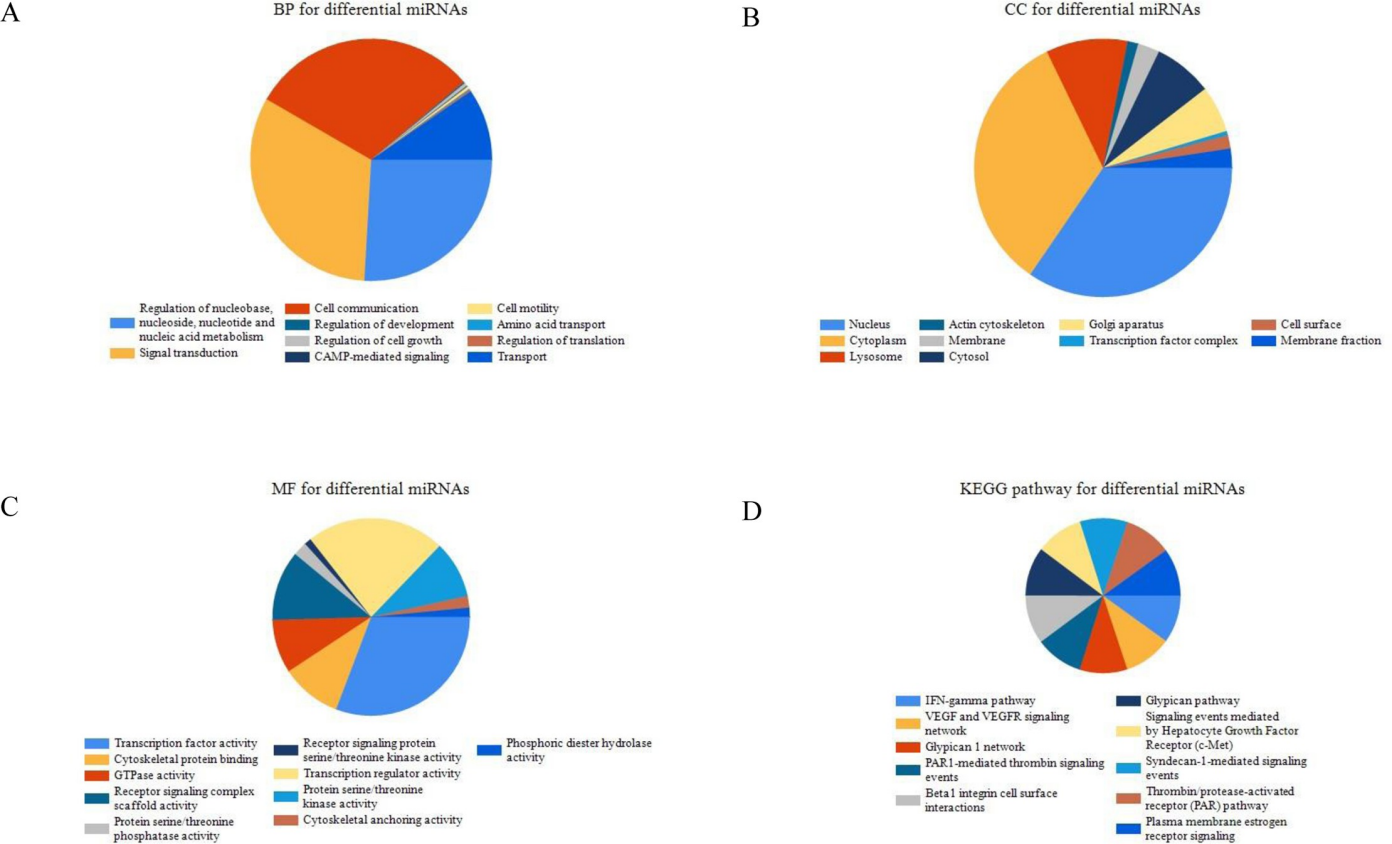
GO and KEGG functional enrichment analysis of DEmiRs. A Biological Process for differential miRNA. B Cellular Component for differential miRNA. C Molecular Function for differential miRNA. KEGG pathway of differential miRNAs.

In order to study the biological function of DEmiRs, GO and KEGG functional enrichment analysis were conducted. The results showed that these DEmiRs were significantly enriched in various functional characteristics. GO analysis results showed that in terms of biological process, DEmiRs are mainly enriched in cell communication, signal transduction and Regulation of nucleobase, nucleoside, nucleotide and nucleic acid metabolism (Fig 2A); that cellular components are mainly enriched in Cytoplasm, Nucleus Etc. (Fig 2B); that molecular functions are mainly enriched in Transcription regulator activity, Transcription factor activity, etc. (Fig 2C). KEGG functional enrichment analysis showed that DEmiRs are more uniformly enriched in tumor-related pathways, such as Beta1 integrin cell surface interactions, Glypican pathway and *VEGF* and *VEGFR* signaling network (Fig 2D).

### 3.3 PPI network construction and module selection

**Fig 3:**
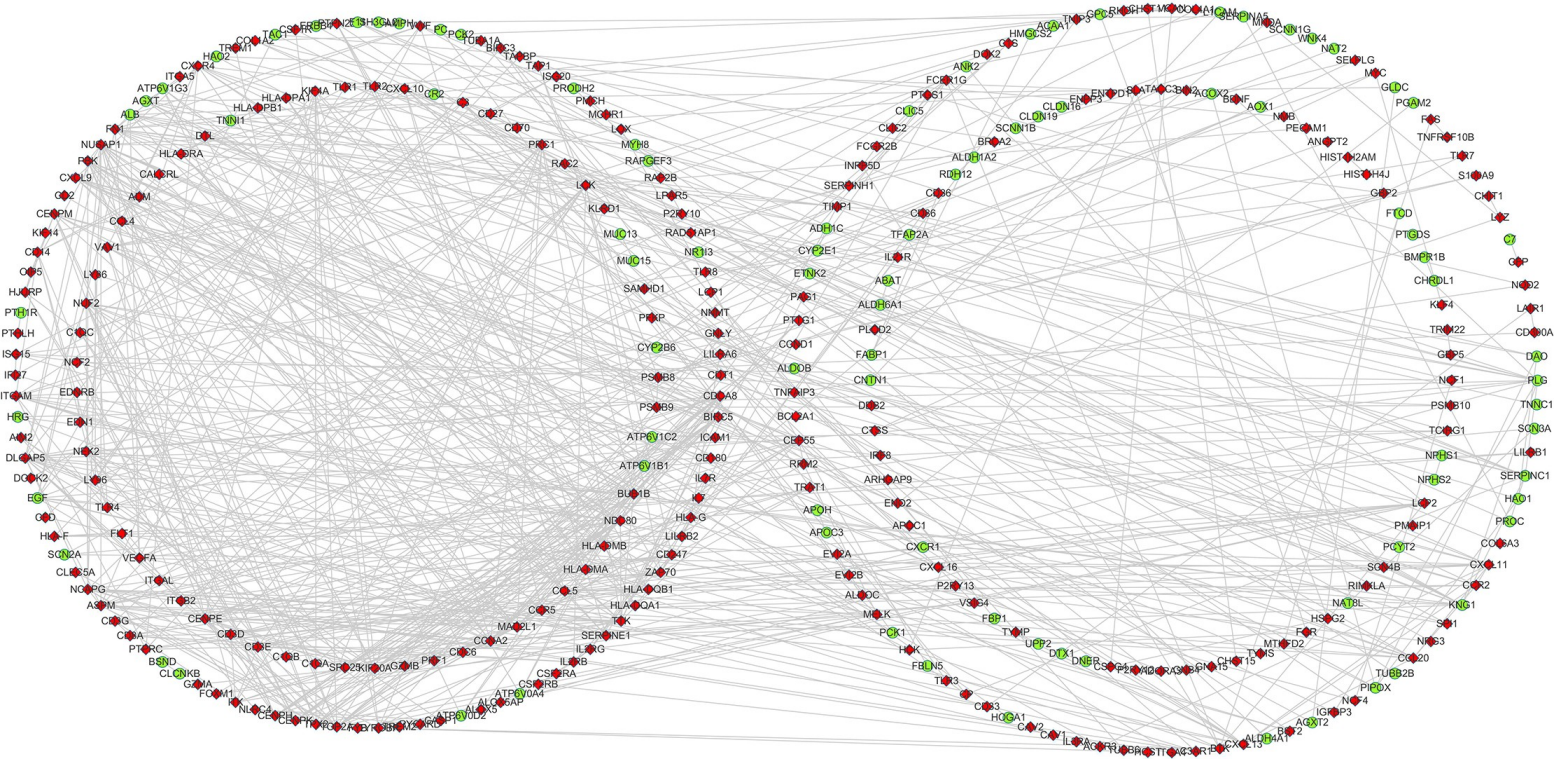
Protein-protein interaction regulatory network of DEGs in CCRCC. Nodes represent DEGs. Red nodes indicate upregulated DEGs and green nodes indicated downregulated DEGs. Edges stand for the regulatory association between any 2 nodes.

**Fig 4:**
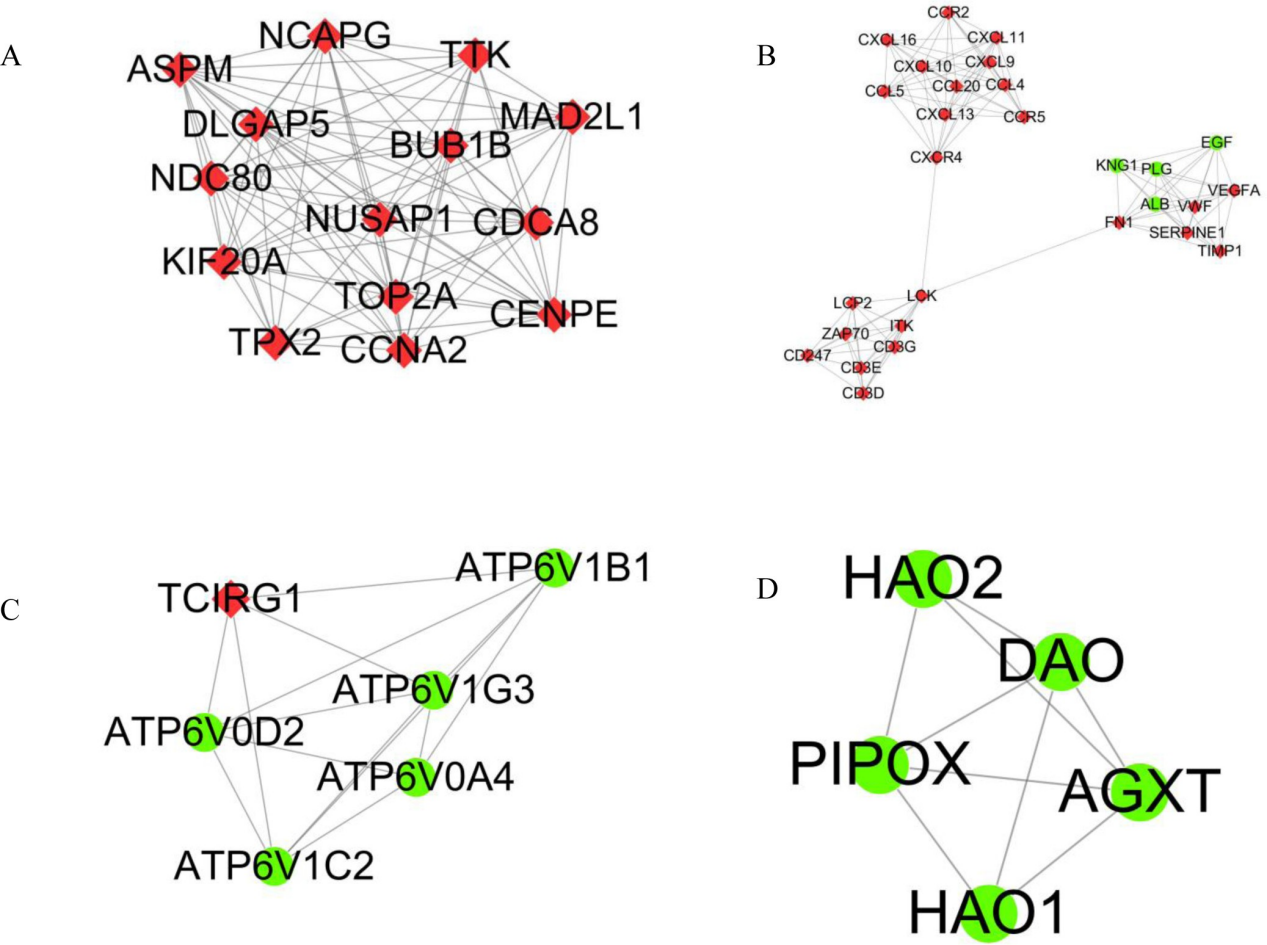
Top 4 modules in the protein‑protein interaction network for DEGs. A Module 1. B Module 2. C Module 3. D Module 4. Nodes represent DEGs. Edges stand for the regulation association between any 2 nodes. Red and green nodes represent upregulated and downregulated genes.

**Fig 5:**
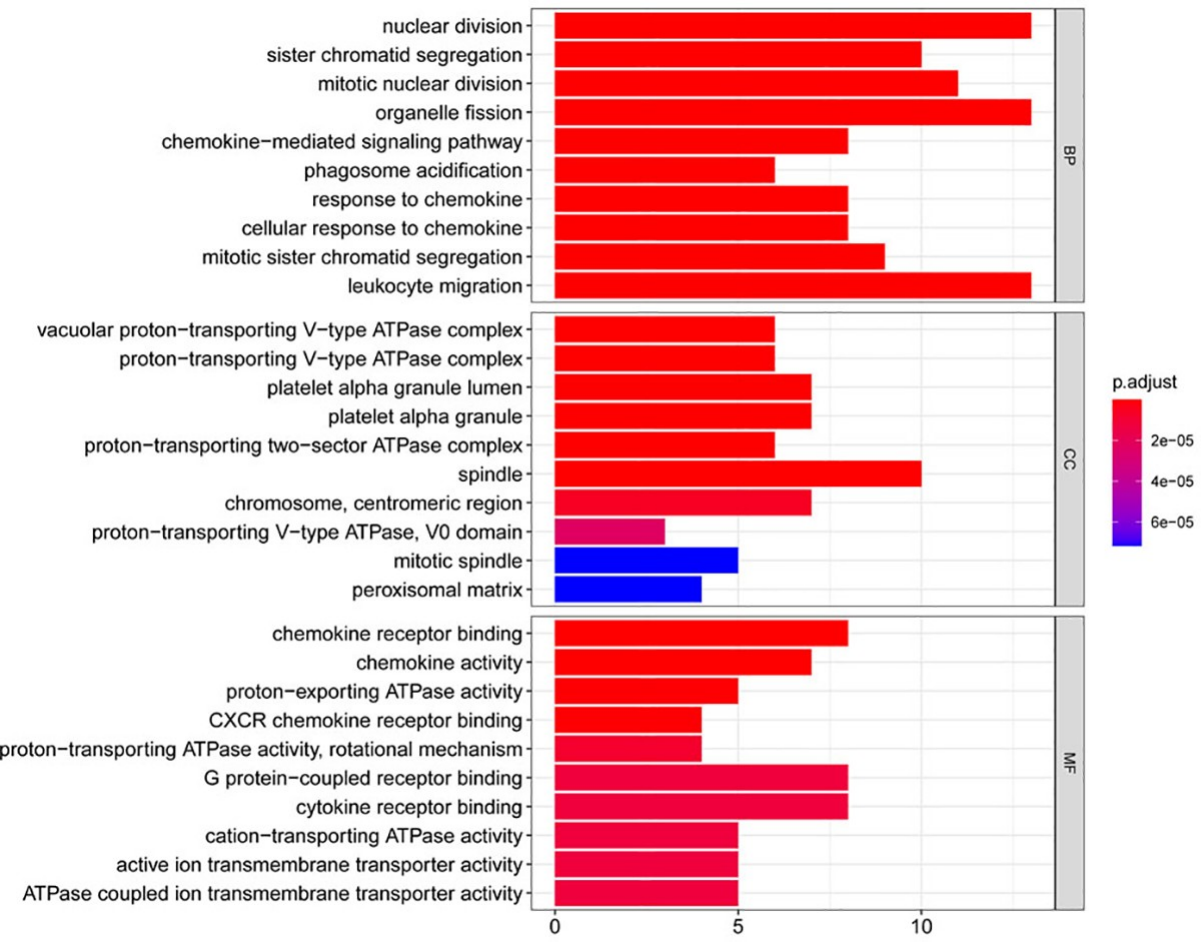
Gene ontology analysis of DEGs in CCRCC.

**Fig 6:**
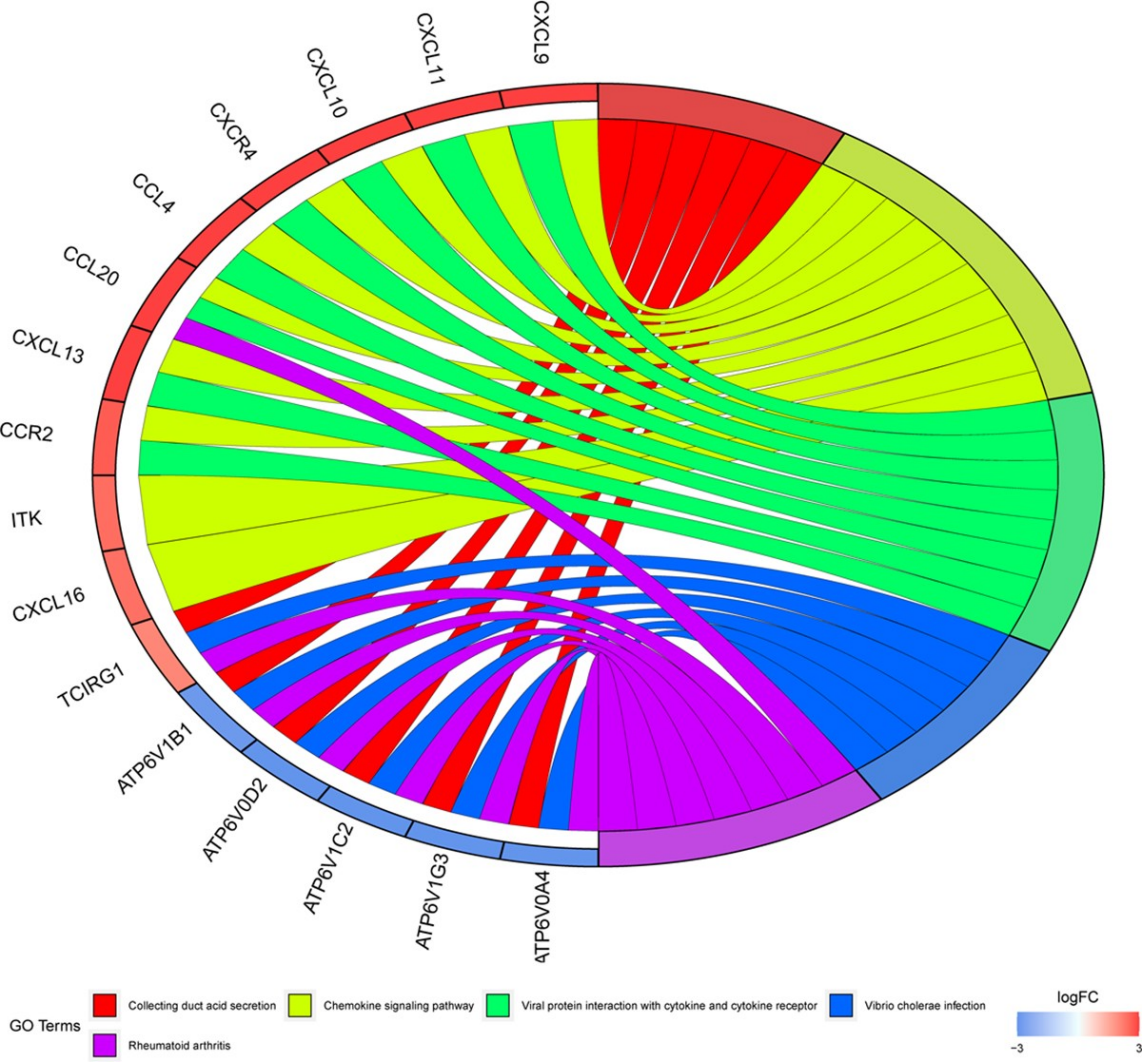
KEGG pathway analysis of DEGs in CCRCC.

Using string online tool to generate PPI network of DEGs, confidence Score ≥ 0.95, the network was imported into the software of Cytoscape. The network included 336 nodes and 706 edges, including 247 up-regulated genes and 89 down-regulated genes (Fig 3). Then the cyclotypeMCODE was used for further analysis. The MCODE analysis showed that there were 16 available modules in total, and the first four modules with the highest score were selected for analysis (Fig 4), including 47 genes in total. Then GO (Table 1 and Fig 5) and KEGG enrichment analysis (Table 2 and Fig 6) were carried out for the DEGs between these modules. Go enrichment analysis results showed that in the aspect of biological process, the DEGs in the module were mainly enriched in the aspects of nuclear division, sister chrome segregation, mitotic nuclear division,etc; the cellular component was mainly enriched in the aspects of vacuum proton transporting V-type ATPase complex, proton transporting V-type ATPase complex, platelet alpha grain lumen, etc; molecular function was mainly enriched in the aspects of chemokine receiver binding, chemokine activity proton exporting ATPase activity, etc. KEGG pathway enrichment analysis results showed that DEGs is mainly enriched in those aspects, for example,collecting duct acid secret, chemokine signaling pathway, vital protein interaction with cytokine and cytokine receiver, etc.

**Table 1:** Gene ontology analysis of DEGs in CCRCC.

ONTOLOGY	ID	Description	GeneRatio	BgRatio	pvalue	p.adjust	qvalue
BP	GO:0000280	nuclear division	13/46	407/18670	1.10E-11	1.02E-08	6.65E-09
BP	GO:0000819	sister chromatid segregation	10/46	189/18670	2.65E-11	1.02E-08	6.65E-09
BP	GO:0140014	mitotic nuclear division	11/46	264/18670	3.16E-11	1.02E-08	6.65E-09
BP	GO:0048285	organelle fission	13/46	449/18670	3.75E-11	1.02E-08	6.65E-09
BP	GO:0070098	chemokine-mediated signaling pathway	8/46	88/18670	3.97E-11	1.02E-08	6.65E-09
BP	GO:0090383	phagosome acidification	6/46	28/18670	5.77E-11	1.23E-08	8.05E-09
BP	GO:1990868	response to chemokine	8/46	97/18670	8.78E-11	1.40E-08	9.19E-09
BP	GO:1990869	cellular response to chemokine	8/46	97/18670	8.78E-11	1.40E-08	9.19E-09
BP	GO:0000070	mitotic sister chromatid segregation	9/46	151/18670	9.94E-11	1.41E-08	9.26E-09
BP	GO:0050900	leukocyte migration	13/46	499/18670	1.38E-10	1.64E-08	1.08E-08
CC	GO:0016471	vacuolar proton-transporting V-type ATPase complex	6/46	17/19717	1.39E-12	1.84E-10	8.96E-11
CC	GO:0033176	proton-transporting V-type ATPase complex	6/46	26/19717	2.55E-11	1.69E-09	8.20E-10
CC	GO:0031093	platelet alpha granule lumen	7/46	67/19717	1.83E-10	8.04E-09	3.91E-09
CC	GO:0031091	platelet alpha granule	7/46	91/19717	1.63E-09	5.05E-08	2.46E-08
CC	GO:0016469	proton-transporting two-sector ATPase complex	6/46	51/19717	1.91E-09	5.05E-08	2.46E-08
CC	GO:0005819	spindle	10/46	347/19717	5.82E-09	1.28E-07	6.23E-08
CC	GO:0000775	chromosome, centromeric region	7/46	193/19717	2.99E-07	5.64E-06	2.75E-06
CC	GO:0033179	proton-transporting V-type ATPase, V0 domain	3/46	10/19717	1.41E-06	2.33E-05	1.13E-05
CC	GO:0072686	mitotic spindle	5/46	109/19717	5.39E-06	7.17E-05	3.49E-05
CC	GO:0005782	peroxisomal matrix	4/46	51/19717	5.98E-06	7.17E-05	3.49E-05
MF	GO:0042379	chemokine receptor binding	8/46	66/17697	5.63E-12	8.39E-10	4.86E-10
MF	GO:0008009	chemokine activity	7/46	49/17697	3.94E-11	2.93E-09	1.70E-09
MF	GO:0036442	proton-exporting ATPase activity	5/46	30/17697	1.29E-08	4.85E-07	2.81E-07
MF	GO:0045236	CXCR chemokine receptor binding	4/46	11/17697	1.30E-08	4.85E-07	2.81E-07
MF	GO:0046961	proton-transporting ATPase activity, rotational mechanism	4/46	22/17697	2.82E-07	8.41E-06	4.87E-06
MF	GO:0001664	G protein-coupled receptor binding	8/46	280/17697	5.51E-07	1.21E-05	7.01E-06
MF	GO:0005126	cytokine receptor binding	8/46	286/17697	6.46E-07	1.21E-05	7.01E-06
MF	GO:0019829	cation-transporting ATPase activity	5/46	66/17697	7.53E-07	1.21E-05	7.01E-06
MF	GO:0022853	active ion transmembrane transporter activity	5/46	67/17697	8.12E-07	1.21E-05	7.01E-06
MF	GO:0042625	ATPase coupled ion transmembrane transporter activity	5/46	67/17697	8.12E-07	1.21E-05	7.01E-06

**Table 2:** KEGG pathway analysis of DEGs in CCRCC.

ID	Description	GeneRatio	pvalue	p.adjust	qvalue	geneID	Count
hsa04966	Collecting duct acid secretion	6/37	1.72E-09	1.75E-07	1.25E-07	ATP6V1C2/ATP6V1B1/TCIRG1/ATP6V0A4/ATP6V0D2/ATP6V1G3	6
hsa04062	Chemokine signaling pathway	10/37	8.16E-09	4.16E-07	2.97E-07	CCL4/ITK/CXCL10/CXCL16/CXCL9/CXCR4/CXCL11/CCR2/CCL20/CXCL13	10
hsa04061	Viral protein interaction with cytokine and cytokine receptor	8/37	1.24E-08	4.21E-07	3.00E-07	CCL4/CXCL10/CXCL9/CXCR4/CXCL11/CCR2/CCL20/CXCL13	8
hsa05110	Vibrio cholerae infection	6/37	8.53E-08	2.18E-06	1.55E-06	ATP6V1C2/ATP6V1B1/TCIRG1/ATP6V0A4/ATP6V0D2/ATP6V1G3	6
hsa05323	Rheumatoid arthritis	7/37	1.71E-07	3.49E-06	2.49E-06	CCL20/ATP6V1C2/ATP6V1B1/TCIRG1/ATP6V0A4/ATP6V0D2/ATP6V1G3	7
hsa05120	Epithelial cell signaling in Helicobacter pylori infection	6/37	6.59E-07	1.12E-05	7.98E-06	ATP6V1C2/ATP6V1B1/TCIRG1/ATP6V0A4/ATP6V0D2/ATP6V1G3	6

### 3.4 miRNA-mRNA interactions analysis and visualization

**Fig 7:**
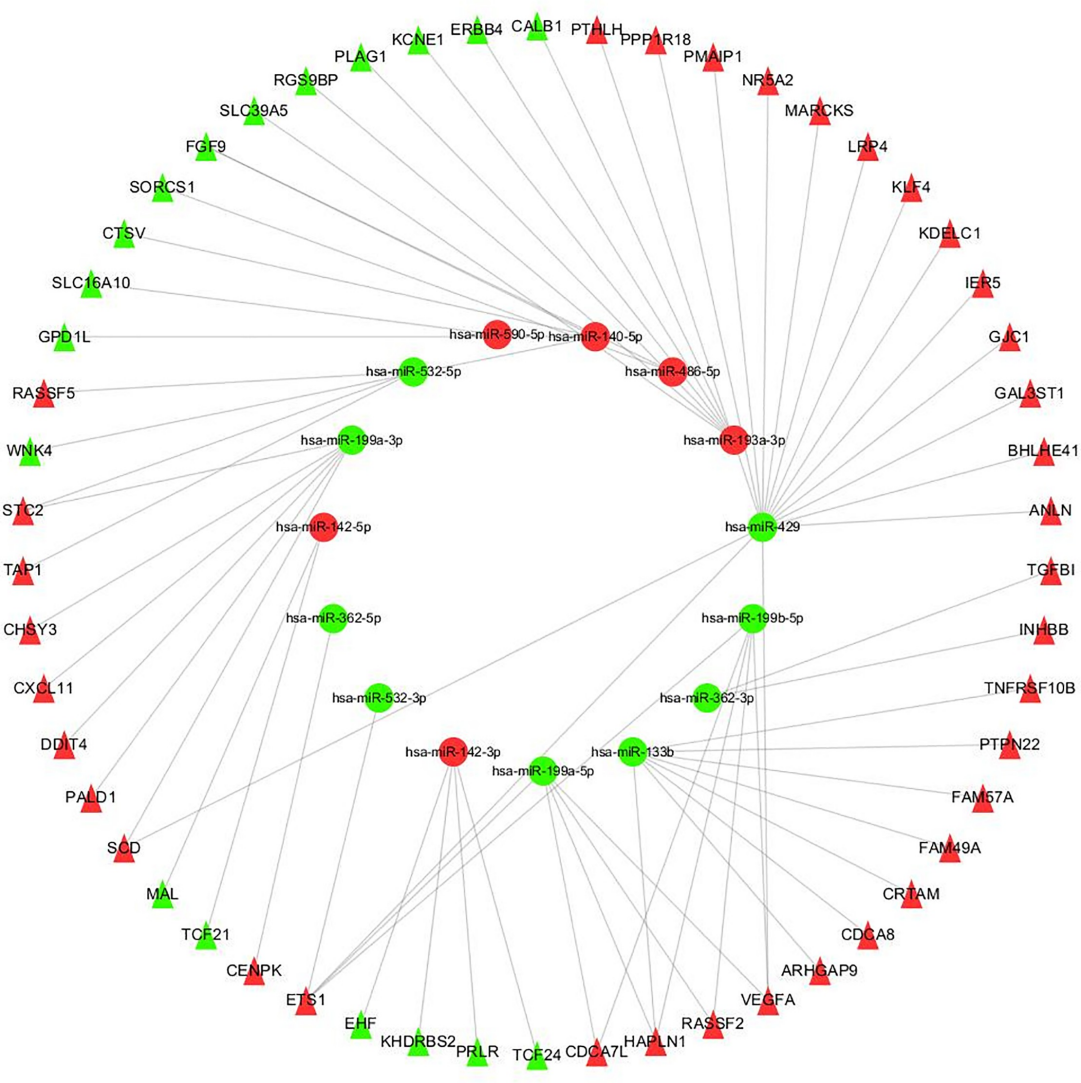
Regulation network of DEmiRs and DEGS. The oval node represents miRNA, the triangle node represents mRNA, the line represents the targeted regulation relationship between them, the green represents the downregulation of miRNA or mRNA in CCRCC samples, and the red represents the upregulation of miRNA or mRNA in CCRCC samples.

By using FunRich software, the target genes of DEmiRs was predicted and a total of 2070 target genes were obtained. The targeted regulatory relationship between DEmiRs and DEGS was obtained with Perl (V5.30.0). The subsequent steps involved uploading this control relationship to Cytoscape_3.2.1 software for visual processing, and constructing the control network of DEmiRs and DEGS (Fig 7). In this control network, a total of 69 nodes and 66 edges were included. The elliptical node represents DEmiRs, the triangular node represents DEGS, and the connection represents the targeted regulatory relationship between the two. Green represents miRNA or mRNA down-regulation in CCRCC samples, and red represents miRNA or mRNA up-regulation in CCRCC samples.

### 3.5 Survival analyses

**Fig 8:**
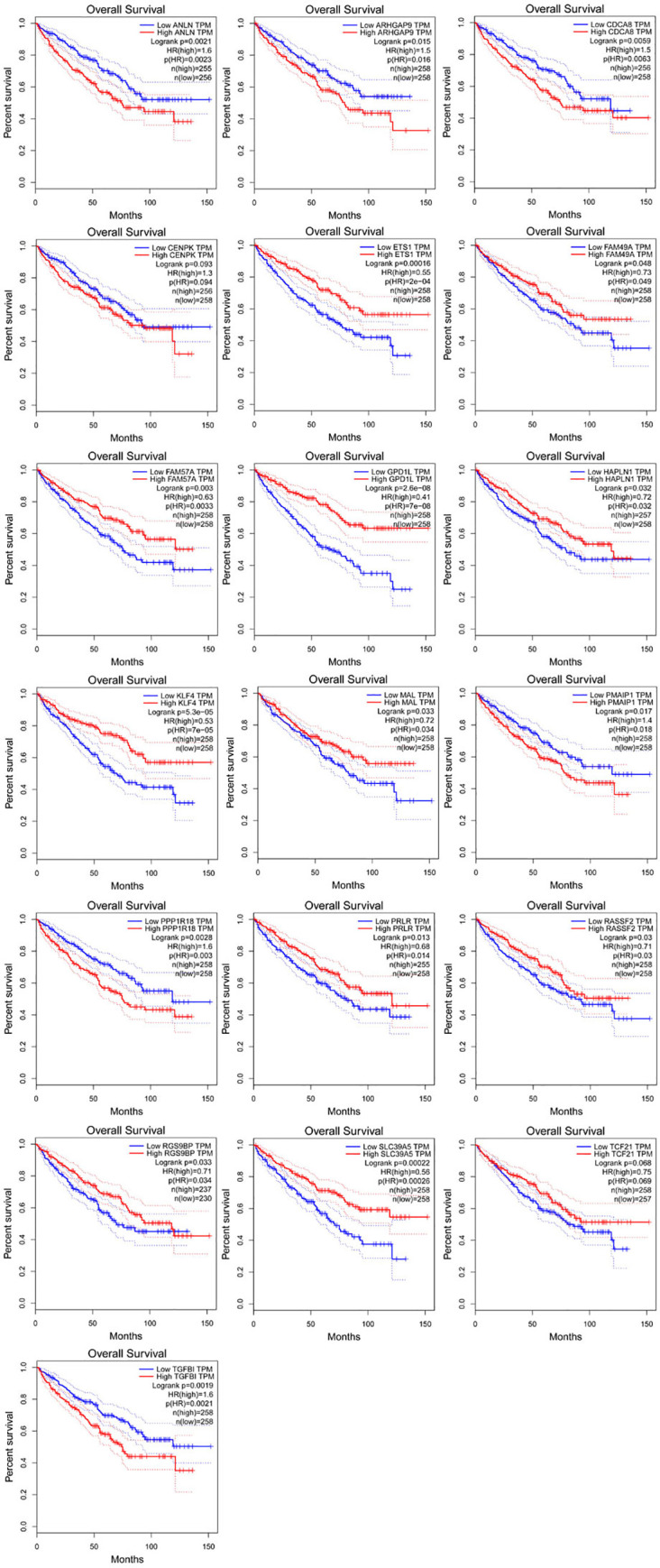
Nineteen differentially expressed mRNAs were associated with survival in CCRCC patients by using the Kaplan‐Meier curve. A, ANLN; B, ARHGAP9; C, CDCA8; D, CENPK; E, ETS1; F, FAM49A; G, FAM57A; H, GPD1L; I, HAPLN1; J,KLF4; K, MAL; L, PMAIP1. M, PPP1R18;N, PRLR;O, RASSF2;P, RGS9BP;Q, SLC39A5;R, TCF21;S, TGFB.

In order to further verify the characteristics of DEGS in the network and its correlation strength with survival, Cox regression analysis, Kaplan-Meier curve and log-rank analysis were adopted to study more about the survival of DEGS in the network. The results showed that a total of 19 DEGS were ultimately deemed to have significant differences in the survival status of CCRCC patients (Fig 8). These included 13 up-regulated DEGs (*ANLN*, *ARHGAP9*, *CDCA8*, *CENPK*, *ETS1*, *FAM49A*, *FAM57A*, *HAPLN1*, *KLF4*, *PMAIP1*, *PPPIR18*, *RASSF2*, *TGFB1*), and six down-regulated DEGS (*GPD1L*, *MAL*, *PRLR*, *PGS9BP*, *SLC39A5*, *TCF21*). Through network topology analysis, it was found that *ETS1* and *HAPLN1’s* degree are the highest, that *ETS1* is regulated by *hsa-miR-199a-5p*, *hsa-miR-199b-5p*, *hsa-miR-532-3p and hsa-miR-429*, and that HAPLN1 is controlled by *hsa-miR-133b*, *hsa-miR-199a-5p* and *hsa-miR-199b-5p* regulation.

## 4 Results and discussion

Renal cell carcinoma is one of the most common malignant tumors in the urinary system, and renal clear cell carcinoma is the most common histological type. Early-stage renal cell carcinoma patients have a higher cure rate, with a 5-year survival rate of more than 90%. However, distant metastasis was found in many patients. Once it was found, and the 5-year survival rate has dropped to 12% [18]. Therefore, there is an urgent need to find out the sensitive early biomarkers. Now RNA sequencing technology has been used to screen out general genetic changes in tumorigenesis and predict potential biomarkers for prognosis [19]. A single miRNA may be involved in the regulation of target mRNA in different carcinogenic or tumor suppressor pathways, hence, it is of great importance for excavating novel prognostic indicators of CCRCC to analyze the regulatory network of miRNA-mRNA interaction at the system level, which may also contribute to control malignant transformation of CCRCC.

In this study, the mRNA and miRNA microarrays of CCRCC patients were analyzed to assess the changes in genes and miRNAs of renal clear cell carcinoma, with a total of 90 DEmiRs (45 up-regulated and 45 down-regulated) and 1309 DEGs (718 Up-regulation, 591 down-regulation. The study found that they have a clear difference in GO functional enrichment analysis after in-depth analysis of the two functional and pathway enrichment. In pathway enrichment analysis, DEmiRs are mainly enriched in Beta1 integrin cell surface interactions, Glypican pathway and *VEGF* and *VEGFR* signaling network, DEGs are mainly enriched in collecting duct acid secretion, Chemokine signaling pathway, viral protein interaction with cytokine and cytokine receptor related pathways, integrin is a transmembrane heterodimer, mainly plays the role of cell adhesion, connecting extracellular matrix and intracellular actin skeleton [20]. Studies have shown that in tumor cells, vascular endothelial growth factor receptor (*VEGFR*) can interact with integrin, thereby activating integrin signal transduction [21]. Now, it is evident that the *VEGF-A* gene and angiogenic growth factors are involved in the development of CCRCC. Many researchers have also begun to focus on the biological research of *VEGFs* and *VEGFR1-3* and the intricate angiogenesis pathway [22]. Glypican and Glypican 1 network are also significantly enriched in pathways. Glypican 1 and other glypicans belong to the heparan sulfate proteoglycan family, which mainly regulates growth factors [23]. Studies have shown that in some cancers, they are highly expressed and are involved in the development of certain cancers. It is reported that Glypican is expected to become a new biomarker in the field of cancer [24]. Glypicans, as the core protein of glycocalyx, are involved in promoting integrin aggregation and growth factor signaling [25]. Therefore, it is guessed that the occurrence and development of CCRCC are closely related to the above pathways. In the pathway enrichment analysis of DEGs, the most significant pathway of enrichment is Chemokine signaling pathway, and chemokines belong to the large family of chemokines. So far, 48 chemokines have been identified. Chemokine CXC subfamily 13 (CXCL13), also known as B cell-attracting chemokine-1 (BCA-1), is a member of CXC-chemokine family. It has been shown that the tissue damage and hypoxic environment exacerbate the progression of prostate cancer, which is achieved by inducing the expression of CXC13 in tumor myofibroblasts [26]. Other studies also indicated that CXCL13 has a good diagnostic and prognostic value for renal clear cell carcinoma [27]. Therefore, chemokine signaling pathway also plays an important role in renal cell carcinoma, and its molecular mechanism needs further study.

In order to further screen the target gene, the target genes of differentially expressed miRNAs were predicted. We took the intersection between them the target regulatory relationship between DEmiRs and DEGs were obtained through Perl (v5.30.0) through Perl (v5.30.0), then we constructed a miRNA-mRNA regulatory network by using Cytoscape_3.2.1.Finally, we analyzed the overall survival of DEGs in the network, among which 19 DEGs were significantly related to the overall survival rate of CCRCC. Through topology analysis, it was found that E26 transformation specificity-1 (*ETS1*) and Hyaluronan and proteoglycan link protein 1 (*HAPLN1*)’s degree are the highest, and these two core genes are highly expressed in CCRCC patients, besides, *ETS1* is affected by *hsa-miR-199a-5p*, *hsa-miR-199b-5p*, *hsa-miR-532-3p and hsa-miR-429* regulated, *HAPLN1* is regulated *by hsa-miR-133b*, *hsa-miR-199a-5p and hsa-miR-199b-5p* simultaneously. As we all know, miRNAs bind to the 3'-UTR of target genes to reduce the protein amount through inhibiting the translation process or promoting the degradation of corresponding mRNA [28]. *ETS1* is a member of ETS transcription factor family. Studies have shown that as an oncogene, *ETS1* is highly expressed in a variety of solid tumors, including lung cancer, colorectal and squamous cancer, breast cancer, ovarian cancer and cervical cancer, etc [29,30]. The high expression of *ETS1* is associated with a poor survival prognosis, which is consistent with our results. Previous studies have shown that ETS1 can directly bind to a special region of the miR-532-5p promoter and inhibit its transcription. As an oncogene of renal cell carcinoma, ETS1 is significantly associated with poor survival in large cohort specimens of renal cell carcinoma [31]. In this study, it was also found that *hsa-mir-199a-5p*, *hsa-mir-199b-5p*, *hsa-mir-532-3p and hsa-mir-429* regulate the expression of *ETS1* at the same time. Therefore, the effect of these four miRNA gene expressions on the expression of *ETS1* in renal cancer tissues and cell lines deserves further study. *HAPLN1* is another gene with the most significant difference, and its high expression is also significantly related to the survival prognosis of CCRCC patients. *HAPLN1* is negatively regulated by *hsa-miR-133b*, *hsa-miR-199a-5p and hsa-miR-199b-5p*. *HAPLN1*, known as cartilage connexin, is expressed in various tissues such as fetal cerebral cortex [32], fetal and adult heart tissue, etc [33]. Alla V et al. [34] found that overexpression of *HAPLN1* and its SP and IgV domains increased the tumor igenicity of mesothelioma. Sihem Mebarki et al. [35] found that knockout of the *HAPLN1* gene downregulated the expression of key markers of liver progenitor cells, such as *Snail*, *LGR5*, *type IV collagen*, *and α-SMA*. Through bioinformatics analysis, Zengzeng Wang et al. [36] also found that hapln1, *hsa-mir-204 and hsa-mir-218* can be used as biomarkers of renal clear cell carcinoma, which further verified our prediction. In this study, the regulatory relationship and mechanism of hapln1, *hsa-mir-133b*, *hsa-mir-199a-5p and hsa-mir-199b-5p* in CCRCC need further study.

In recent years, more and more studies have shown that miRNA plays an important role in inducing complex human diseases [37,38]. Large scale experiments are expensive and time-consuming to explore miRNA disease association. Therefore, researchers try to construct complex networks on the basis of existing biological databases, establish the calculation model of potential miRNA disease association, screen out the most likely potential association, and then conduct small-scale verification through biological experiments, which plays an important role in the study of miRNA disease association. Shi Identified the potential association between miRNA and disease genes by using the association between miRNA targets and disease genes in the protein-protein interaction (PPI) network [39]. Chen [40] calculated the prediction score of miRNA disease pair by combining miRNA similarity and disease similarity, and proposed the induction matrix completion model (IMCMDA) for miRNA disease association prediction. By using Gaussian spectral kernel similarity method to deal with miRNA similarity and disease similarity, a miRNA disease association prediction (MDHGI) calculation model was proposed [41]. In 2019, Chen et al. [42] proposed a miRNA disease association prediction (EDTMDA) calculation method based on decision tree integration. The three calculation models above can predict the potential relationship between miRNA and disease from different perspectives, and promote the understanding of the pathogenesis of complex human diseases, such as cancer. With the development of bioinformatics and sequencing technology, genomics, transcriptomics, proteomics, metabonomics and other omics data research continues to deepen, the integration of multi omics data to achieve efficient cancer biomarker recognition algorithm, find more valuable cancer biomarkers, and provide targeted experimental exploration for researchers will be one of the direction of future researches.

In conclusion, with method of bioinformatics, five miRNAs (*hsa-mir-199a-5p*, *hsa-mir-199b-5p*, *hsa-mir-532-3p and hsa-mir-429*) and two key genes (*ETS1 and HAPLN1*) were found to be the potential biomarkers for the prognosis of patients with CCRCC. However, it is worth noting that the network of miRNA- mRNA interactions is complex, and more scientific exploration are needed to confirm our findings and verify their clinical application potential in improving the survival and prognosis of patients with CCRCC.

## Supporting information

S1 Table GSE16441-GPL8659_series_matrix. (XLS)

S2 Table GSE66270_series_matrix. (XLS)

S3 Table The differentially expressed miRNAs. (XLS)

S4 Table The differentially expressed mRNAs. (XLS)

S5 Table Protein-protein network (PPI) based on the DEmRNAs with a combined score was ≥0.95. (XLS)

S6 Table The miRNA target genes predicted by Funrich 3.1.3. (XLS)

S7 Table The regulatory network of miRNA target genes and differentially expressed mRNAs (DEGS). (XLS)
